# A Remarkable Extra Uterine Case

**Published:** 1810-06

**Authors:** 

**Affiliations:** Andover


### *A remarkable extra* UTERINE CASE.

By Dr. Osgood, *of Andover.*

[ Read before the Massachusetts Medical Society. ]

A. B. of a lax and irritable habit, was married at twenty-two years of age, from which time to the expiration of two years she had two children. From the weakness of her constitution she suffered more than usual during the months of gestation, and remained both times in a weak state for two months after delivery, and was able to give suck to her children not more than five months. In the 25th year of her age, in October, 1784, she became pregnant a third time. She had no complaints in November and December but such as she had been accustomed to during the weeks of conception; but in January, 1785, she complained of an unusual lameness in and about the loins, and particularly the left hip. This lameness gave her but little apprehension of evil, and she made no application to me till the 13th of February, when she was attacked with with those pains which usually precede an abortion. Having inquired what had befallen her the preceding day (for it was evening when I was called) I was informed that she had walked hastily on the ice, and that she had been greatly alarmed by the news that two of her neighbours were thought to be dying. I supposed that these causes were sufficient with her to produce a miscarriage, unless prevented by the assistance of art. I immediately opened a vein, and, after plentiful bleeding, ordered her rest and gave her freely of opiates. These had the desired effect; and the next morning she had no complaints that threatened an abortion, but her lameness was much increased, and she complained of pain in her left side just below the short ribs, and extending to her stomach, together with some uneasiness. I recommended rest and gave her sedatives and antispasmodics.

The 20th of February, she was suddenly seized with pains in her stomach and side: I again had recourse to venesection, and gave opiates with sal succin.; and after extreme pain for six hours, she fell into a swoon. Such paroxysms of distress recurred often, nor was she a day free from pain and uneasiness in her left side, frequently accompanied with excessive vomitings, and fever with costiveness. I endeavoured to give remedies according to the nature of her complaints, mild eccoprotics, antiphlogistics, and sedatives. Such were her complaints till the 13th of March, when suddenly, without pain preceding (for her pains were generally severe before her vomitings) she vomited nearly half a pint of pus, without any warning, nor was there any mixture of food with it, though she had breakfasted but a few minutes before. Her distress was extreme immediately upon this discharge, beyond every thing she had experienced before, and accompanied with a sudden and great debility; indeed it appeared, that now the period of her sufferings was arrived, and that death would shortly close the scene. But Heaven ordained otherwise, and she was to live, to give an example how great sufferings a feeble constitution can bear. She continued to vomit through the day, though very languid, and the remaining discharges from her stomach had some sprays of pus and blood. The next day she was free from pain, but her soreness and languor were great: sore mouth, and other symptoms of a depraved state of the juices appeared; and an unnatural heat, with a weak and quick pulse, gave me much uneasiness, especially as the pain in the left side returned, but not with that severity as before the purulent discharge, discharge. But there was a sensible enlargement just below the short ribs, greater than we could suppose was occasioned by the fœtus, so early in pregnancy, though the motion of the fœtus was sensibly perceived by the patient, and I could plainly distinguish its motions by applying my hand on the left side of the abdomen. The pain, sore mouth, and fever, continued till the 13th of April, when she was attacked with nausea, but did not vomit; but a diarrhœa followed her for that day with appearances of pus mixt with the fæces. I consulted Drs. Baylies and Perry, and we fully believed that the diarrhœa was occasioned by the discharge of another abscess, as the pains in the side abated, and she continued free from extreme distress the remainder of April, and till the latter part of May; during which time her appetite returned, and she gained as much strength as to sit in her chair an hour at a time, though she could not stand erect, nor walk without assistance, nor could she lay on her right side, and was almost every day attended with a difficulty of respiration, which lasted from one to two hours, which I considered as an hysteric affection of the lungs, as she generally found relief by fœtid and carminative remedies. But as my patient was much better, and the month of June was near, we hoped that at the expiration of her pregnancy her trials would be at an end; but the month of June seemed but the beginning of her trials, for she was then confined to a bed of pain and distress, cramps and convulsions, and was never off it till the last of October. The first of June she was attacked with severe pain in her left side, and just below the sternum, accompanied with vomitings and spurious pains, resembling parturition pains, and continued every day, though not equally severe, till the 20th of June, when her pains were such as usually attend labour, or rather were grinding pains not any wise forcing, but they continued fourteen hours very regular and severe; I frequently examined her vagina, but could make no discovery that labour was approaching. But as she had repeatedly, for twenty days, had pains similar, to no purpose, and I had given opiates, I was determined to let her pains go on and only support nature, till nature being exhausted the pains almost ceased, and I then gave an opiate. She had no more pains from that time that gave me any encouragement that she would be delivered. The 24th she informed me that the last night she had no rest nor pain, but was much convulsed through the night, and said that her child was dead. I endeavoured to persuade her out of that opinion, opinion, as the want of motion for a few hours could not be an infallible symptom of the death of the fœtus; but she was not to be persuaded: I was however in a few days convinced that it was really the case, as no motion from that time was perceivable; and her breasts which had been turgid became flabby, and she was attacked with faintness, which continued an hour notwithstanding a free admission of air, and a free use of vinegar, volatiles and cordials. This faintness returned every day for three weeks, and lasted nearly the same time, and it appeared sometimes as if she would fall into a total deliquium. When she was not faint, she was a great part of the time in great distress, and the abdomen for some time appeared rather to increase than lessen; but whether the enlargement was occasioned by flatulencies in the intestines, or was in consequence of the death of the fœtus, I could not determine. The 20th of July she was attacked with a uterine hæmorrhage, which was moderate and accompanied with bits of a fleshy substance, which I then considered as parts of the placenta; and, as she had some slight pains in the region of the uterus, I once more hoped that a delivery might be effected, especially as the os tincæ was a little dilated. This discharge continuing for several days, with the above appearances, and no fœtor attending them, nor any further enlargement of the os tincæ, nor any pains that might be considered as parturition pains, I began to think seriously that the fœtus was not in the uterus. I wrote to Dr. Appleton, of Boston, to my honoured father at Andover, and called in the advice of Dr. Baylies; they all agreed that the fœtus was not in the uterus; and as palliatives and a supporting diet was what I had recommended for a long time, they advised the continuance of the same course.

On the 28th of July, she was attacked with a distressing cramp, which was severe beyond description; the paroxysms continuing from four to eight hours, accompanied with severe convulsions. The paroxysms at first were not very frequent; but the latter part of August and the month of September, they returned almost every day, and nothing but opium and the warm bath, with frictions, gave any relief: musk, amber, and fœtid gums were tried to no purpose; and finally, she was obliged to take twenty grains of opium in a day. Her disorder was so violent as to produce all the varieties of tetanus, emprosthotonos, opisthotonos, &c. and every muscle was, in turn, affected. Such was the severity of this disorder, that it appeared as if every every paroxysm would put a period to her existence. But as the cold weather advanced, and the size of the abdomen lessened, these severe spasms abated; and after the 5th of October she had no paroxysms of the cramp; but flatulencies of the bowels prevailed; and as there appeared some better prospects, I sought for some effectual means for their removal. Having lately read the history of Mrs. Low, in the American Magazine, printed in Boston, for 1746, and found that great benefit accrued to her from the application of the emplastrum e cymino to the abdominal region, I made the same application, and it afforded her relief beyond my expectations. Such for four months past had been the weakness of her digestive powers, that no solid food could be taken without exciting immediate distress; but by the middle of October she could receive an animal diet, so that by the first of December she rode out, and in January, 1786, had a return of her menses, which were regular till January, 1788, when she became pregnant, and at the expiration of nine months was delivered of a well-grown healthy child. During this last pregnancy and labour she was as well as she ever had been when pregnant, or at the time of delivery. Before her last pregnancy, the tumour occasioned by the fœtus was about the size of the head of a nine-month fœtus, laying to the left of the umbilicus, extending downwards to the pubes. But the first fortnight after her last delivery the tumour was mostly upon her right side, but is now, when standing, directly over the pubes. From these motions it was conjectured that the fœtus was originally in the left Fallopian tube, but that the tube was burst.

She was delivered of a son in November, 1790, and another son in July, 1793, and of a daughter in January 1796; during which time her health was better than I should have expected, nor did the tumour in her left side, during her pregnancies, cause greater uneasiness than in her pregnancy of 1788; nor were her sufferings greater in these several labours than was common in her labours before the tumour in 1785. After the birth of the daughter in 1796, her strength did not return as usual, and her debility was increased by an abortion the latter part of December of the same year. She had abortions every year from that time till the present year 1802, when on the 17th of January, she was delivered of a healthy daughter. The first months of this last pregnancy her complaints were not more than she had commonly experienced in the early early stages of pregnancy. She rode several short journies, and attended to her domestic concerns. About the fifth month, as the enlargement of the uterus pushed the tumour out of the pelvis, the pain and soreness were very distressing, much more than in any preceding pregnancy (except the pregnancy, the supposed cause of the tumour in 1784 and 1785). The uneasiness was confined to the left side, and in the place of the tumour, and every motion from within and without was distressing. These complaints lessened after the seventh month, but the general complaints of a gravid uterus were very distressing, and for two months before the birth of the child she was frequently afflicted with pains, that gave apprehensions of immediate labour. On the evening of the 16th January, labour commenced by the breaking of the membranes, but the pains were not very considerable till the morning of the 17th, when her pains, or rather distress, was constant, and continued without cessation for four hours, and she was then delivered of a full-grown healthy daughter. The hæmorrhage and pains were not so great after labour as she had been accustomed to, and she had no unusual complaints till the evening of the 19th, when she discharged several large grumous clots, then slept and awoke at four o'clock of the 20th with universal distress, and a long continued rigour, which was followed with great heat. By the timely use of sedative and antiphlogistic medicines the violent symptoms abated; but alternate chills and heat, with a tense abdomen, continued. The lochia in common quantity, and the milk appeared at the usual time, but not in usual quantity; and by the sixth day the milk was very trifling, and soon ceased. The left side of the abdomen was much enlarged, and the pain and soreness distressing. The latter part of January aphthæ appeared, and the singultus was distressing. These complaints for a few days were alarming, and threatened immediate dissolution. The 10th of February the heat abated, the aphthæ and hiccups much lessened, and her appetite better; but her strength not increased, and the rigours and paroxysms of fever were as before. February 15th she had several stools, (the first from January 17th without an enema or cathartic) and symptoms no worse till the evening, when the discharges became frequent, and, instead of fæces, uniform pus, which was extremely fœtid, and continued till 10 o'clock of the 16th, when great debility was induced, and I momentarily expected a period to her sufferings ferings and life. After several hours of extreme languor she revived, the tumours lessened, and by the 20th she was recruited to nearly the same strength as before the purulent discharges; the quantity of pus evacuated from the 15th to the 20th was estimated at three pints. From this time it accompanied almost every discharge of fæces, and frequently the discharges were only pus, and very offensive: her bowels were no longer constispated, her appetite better, but by no means regular; and, at all times she complained of an indescribable nauseous taste. From the first of the aphthæ she frequently vomited, and the ptyalism for three weeks was very great; nor did an aphthous state of the fauces leave her. The latter part of April she was able to sit in her chair, and with assistance walked; but her feet swelled, and her appetite lessened. May 4th and 5th, the weather being very warm, she vomited almost every thing she took, which continued till the 13th, then ceased, sore mouth came on and delirium, and she expired on the 22d.

On the 23d, Doctors Bricket, Kittredge and Pearson, were called to dissect and examine the tumour, which had afflicted her for more than seventeen years, and which I supposed the cause of her death. She being a near friend I was not present at the dissection. They informed me, that the tumour extended from the navel, lying on the left side, and into the pelvis. They found the uterus in a sound state, as was the right Fallopian tube: the left Fallopian tube was very greatly enlarged and entire, and they took therefrom the bones of a full-grown fœtus.* The tube adhered to the peritonæum from the upper edge of the os ilium for some considerable distance above. It also adhered to the colon, where there was an aperture, which was the outlet of the pus, which continued to discharge from the 15th February till her death. The tube had thickened with its enlargement, and was found to be nearly half an inch in thickness; the bones of the fœtus had nearly perforated the tube in several places. The ovarium and fimbriæ were obliterated.

* These bones are now in the possession of the Society.

